# cGAS-STING Signaling as a Molecular Bridge Between Inflammation, Ovarian Ageing, and Reproductive Failure

**DOI:** 10.3390/ijms27104559

**Published:** 2026-05-19

**Authors:** Charalampos Voros, Fotios Chatzinikolaou, Georgios Papadimas, Ali Can Gunes, Aristotelis-Marios Koulakmanidis, Ioannis Papapanagiotou, Athanasios Karpouzos, Diamantis Athanasiou, Kyriakos Bananis, Antonia Athanasiou, Aikaterini Athanasiou, Charalampos Tsimpoukelis, Maria Anastasia Daskalaki, Christina Trakatelli, Nikolaos Thomakos, Panagiotis Antsaklis, Dimitrios Loutradis, Georgios Daskalakis

**Affiliations:** 1 Department of Obstetrics and Gynecology, ‘Alexandra’ General Hospital, National and Kapodistrian University of Athens, 80 Vasilissis Sofias Avenue, 11528 Athens, Greece; 2 Laboratory of Forensic Medicine and Toxicology, School of Medicine, Aristotle University of Thessaloniki, 54124 Athens, Greece; 3 Athens Medical School, National and Kapodistrian University of Athens, 15772 Athens, Greece; dr.georgepapadimas@gmail.com (G.P.);; 4 Department of Obstetrics and Gynecology, Faculty of Medicine, Hacettepe University, Ankara 06100, Turkey; 5 King’s College Hospitals NHS Foundation Trust, London SE5 9RS, UK; 6 3rd Department of Internal Medicine, Aristotle University, 54124 Thessaloniki, Greece

**Keywords:** cGAS-STING, ovarian ageing, infertility, granulosa cells, mitochondrial DNA, inflammation, senescence, uterine receptivity, endometriosis, reproductive failure

## Abstract

Infertility and ovarian ageing are increasingly acknowledged as illnesses affected not just by endocrine decline but also by chronic inflammatory stress and mitochondrial dysfunction in the reproductive milieu. The cGAS-STING signalling pathway has emerged as a significant possibility linking these activities. The cGAS-STING pathway, originally defined as a cytosolic DNA-sensing mechanism essential for innate immune defence, is now recognised as a broader modulator of sterile inflammation, cellular senescence, and tissue failure. Experimental reproductive models suggest that the activation of this system may operate as a crucial link between mitochondrial dysfunction, cytosolic DNA accumulation, inflammatory cytokine production, and the progressive decline of ovarian and endometrial function. The activation of cGAS-STING in granulosa cells has been associated with inflammatory signalling and impaired steroidogenic activity.

## 1. Introduction

Infertility is no longer seen just as a condition of hormonal imbalance or structural disturbance. There is increasing interest in the molecular stability of the follicular and endometrial microenvironments. Protecting mitochondrial function, organising DNA inside cells, maintaining redox balance, and regulating inflammatory signals are all aspects that impact the ability to reproduce [1]. Subtle alterations in intracellular signalling networks, mitochondrial function, oxidative stress regulation, and granulosa cell homeostasis may precede clinically detectable reproductive dysfunction. These alterations gradually modify the biological framework of oocyte maturation and implantation [2]. A transition to pro-inflammatory signalling, even without evident disease, is adequate to disturb the meticulously controlled connections between somatic and germ cells. There is a growing interest in mechanisms that detect cellular damage and translate it into coordinated transcriptional responses, particularly those that connect metabolic stress to immunological activation [3].

Ovarian ageing signifies a gradual deterioration in oocyte competency that cannot be exclusively attributed to follicle depletion. Oxidative damage, mitochondrial malfunction, and persistent low-grade inflammation gradually alter the follicular niche. Granulosa cells, which facilitate egg maturation via metabolic and paracrine support, seem to be particularly responsive to these stress signals [4]. The functional deterioration of this cellular compartment has been consistently associated with diminished fertilisation capability and inadequate embryonic development. Alterations in mitochondrial dynamics, such as increased fission, decreased fusion, and impaired mitophagy, lead to the accumulation of dysfunctional organelles and an elevation in reactive oxygen species [5]. The resultant redox imbalance impairs steroidogenesis, intracellular signalling, and energy availability, eventually undermining oocyte developmental competence. Evidence increasingly indicates that ovarian ageing signifies a coordinated deterioration of cellular homeostasis rather than a simple quantitative decline of follicles [6].

The activation of the innate immune system has emerged as a crucial component of this process. Cytosolic DNA-sensing pathways, first recognised in antiviral defence systems, are progressively associated with sterile inflammatory diseases [7]. Under healthy settings, cGAS-STING signalling detects aberrant or exogenous DNA and coordinates temporary immune activation, aiding in intracellular surveillance and host defence. This pathway is associated with antiviral responses, preservation of genomic integrity, and modulation of cellular stress adaptability. Controlled activation contributes to tissue homeostasis and cellular protection during acute stress or injury. Cyclic GMP-AMP synthase (cGAS) and its downstream adaptor, stimulator of interferon genes (STING), collaborate to identify mislocalized DNA and initiate a robust inflammatory response. This pathway is activated without cues from pathogens. For instance, DNA produced under cellular stress serves as a potent trigger. Identifying these signals establishes a paradigm whereby damage-associated molecular patterns, rather than exogenous pathogens, initiate immune response in reproductive organs. The continuous activation of these pathways serves as a fundamental mechanism connecting cellular stress to chronic inflammation in the ovary and endometrium [8].

Experimental findings in ovarian models indicate that this system is inherently present in granulosa cells and swiftly triggered upon exposure to cytosolic DNA mimics, resulting in the production of interferon and the release of inflammatory cytokines. The functional repercussions transcend immune signalling, influencing steroidogenesis and disturbing endocrine equilibrium inside the follicle [9]. Upon activation of the route, the levels of critical enzymes involved in the synthesis of oestrogen and progesterone fluctuate, indicating a direct impact on hormonal balance. Elevated levels of tumour necrosis factor and interleukins foster an inflammatory environment. This impedes the development of follicles and the maturation of oocytes. Recent studies indicate that mitochondrial damage serves as a significant upstream trigger by facilitating the leakage of mitochondrial DNA into the cytoplasm, hence directly activating cGAS-STING signalling and intensifying inflammatory cascades. These findings indicate that granulosa cells function not just as passive responders but also as proactive facilitators of inflammatory signalling within the follicular milieu [10].

Our study examines the emerging function of cGAS-STING signalling as a key mediator linking mitochondrial instability, inflammatory activation, and reproductive decline. The focus is on ovarian ageing, granulosa cell function, and their consequences for infertility and the effectiveness of assisted reproduction. Emphasis is put on the interaction between leaky mitochondrial DNA, innate immune detection, and the creation of a pro-inflammatory milieu that adversely affects reproductive competence. Comprehending these pathways assists researchers in developing novel diagnostics and targeted therapeutics designed to re-establish cellular equilibrium within the reproductive system.

## 2. Biological Background

### 2.1. cGAS-STING Signaling Pathway: Molecular Architecture and Functional Dynamics

Intracellular surveillance systems depend on the ability to distinguish properly compartmentalized nucleic acids from mislocalized or ectopic genetic material. Some scientists think that double-stranded DNA outside of the nucleus or mitochondria is a sign that something is wrong with the cell [11]. Such types of things can happen because of replication stress, faulty DNA repair, unstable mitochondria, or changes in the structure of membranes inside cells. When DNA becomes mislocalized within the cytoplasm, it starts signalling cascades that go beyond normal host defence and affect the fate of the cell [12]. The structure of the nuclear envelope and the stability of the mitochondrial membrane are very important for keeping DNA from being exposed. On the other hand, problems with lamina-associated proteins or changes in chromatin organization make it more likely that DNA will move. Cytoplasmic chromatin fragments are also made when cells are under mechanical stress, when they are damaged by oxidative stress, or when they make mistakes during mitosis. Because even temporary movement can turn on downstream signalling with long-lasting effects, cellular systems are always under pressure to keep genetic material in the right place [13].

Cyclic GMP-AMP synthase directly detects cytosolic DNA and changes shape when it binds. When DNA interacts with it, it changes shape in a way that allows it to act as a catalyst, which makes cyclic GMP-AMP, a second messenger that is very specific for signalling components downstream [13]. This molecule’s formation is an important step in amplification because it lets small amounts of DNA create a long-lasting signal inside cells. The spatial organization of DNA-cGAS complexes makes signal propagation even better. New evidence suggests that dynamic condensates form that concentrate signalling molecules and stabilise activation [14]. Properties of these condensates are in agreement with liquid–liquid phase separation, which may cause the cytoplasm to develop dynamic membraneless compartments. Phase separation augments cGAS activation by increasing the local concentration of DNA and signalling molecules, hence promoting prolonged enzymatic activity and enhancing cyclic GMP-AMP synthesis. Additionally, these condensates may facilitate extended downstream activation of STING-dependent inflammatory pathways and shield signalling complexes from premature destruction. These kinds of condensates are caused by multiple interactions between DNA and protein domains, which allows microenvironments with different phases to form inside the cytosol. The local concentration of substrates and enzymes in these structures makes catalysis more efficient and signalling lasts longer. Different sizes, shapes, and chemical changes in DNA fragments also affect activation thresholds. This suggests that the quality of DNA is just as important as the quantity for pathway engagement [15].

The main job of the stimulator of interferon genes is to convert second messenger signalling into responses downstream. When STING is not active, it is attached to the endoplasmic reticulum membrane in a way that keeps it inactive. When cyclic GMP-AMP binds, it causes a change in structure that encourages oligomerisation and movement toward perinuclear compartments [16]. Moving across membranes inside cells exposes STING to regulatory factors and makes it easier for signalling complexes to form, which are needed for downstream activation. So, trafficking dynamics are very important for determining how strong and how long signalling is. Proper localisation depends on vesicular transport mechanisms and interactions with Golgi-resident proteins [17]. On the other hand, lipid modifications like palmitoylation keep STING stable in signaling-competent domains. Changes in signalling kinetics can happen when intracellular trafficking is disrupted, either by metabolic stress or membrane dysfunction. This can make pathway activation less or more intense. So, how well signals spread depends on how well membrane dynamics and protein modification work together [18].

The recruitment of TANK-binding kinase 1 is an important step in the spread of signals. When this kinase is turned on, it adds a phosphate group to interferon regulatory factor 3, which then dimerises and moves to the nucleus. When nuclear factor kappa B is activated at the same time, it sets off a coordinated transcriptional response that includes the activation of interferon-stimulated genes and pro-inflammatory mediators [19]. Transcriptional output goes beyond the usual immune defence and affects metabolism, stress adaptation, and survival pathways in cells. Cells can respond to damage in different ways depending on the situation because these signals work together. Members of the STAT family and AP-1 complexes are other transcriptional regulators that help fine-tune gene expression profiles based on the type of cell and how long the signalling lasts [20]. Crosstalk between metabolic pathways affects mitochondrial respiration, glycolytic flux, and redox balance, connecting immune activation to the overall health of the cell. So, long-term transcriptional activation changes how cells work at many levels, with effects that go beyond just the immediate inflammatory response.

Mitochondrial integrity is very important for controlling pathway activation. When mitochondrial homeostasis is disrupted, the membranes become more permeable, which makes it easier for mitochondrial DNA to escape into the cytosol. Mitochondrial DNA has unique structural features that make it easier to activate intracellular DNA sensors [21]. These include less methylation and a higher risk of oxidative modification. Changes in mitochondrial dynamics, especially too much fission and poor mitophagy, make it more likely that DNA will be released and keep signalling going over time. Interactions between mitochondrial membrane channels and the formation of pores caused by stress also affect how much DNA leaks out [5]. Proteins like VDAC1 and parts of permeability transition pores help make temporary or permanent openings that let mitochondrial contents escape. If you do not get rid of damaged mitochondria quickly, they build up and keep sending out pro-inflammatory signals. The combined effects of mitochondrial instability make it a constant cause of intracellular DNA sensing.

Long-term activation of this signalling pathway changes how cells behave and pushes them toward a senescent phenotype. Stressed cells build up cytoplasmic DNA fragments, which keep signalling going and cause the production of inflammatory mediators to continue. The resulting secretory profile changes how cells talk to each other and helps remodel tissues at the tissue level [22]. The effects go beyond the organization of the extracellular matrix, paracrine signalling, and the recruitment of immune cells and make a microenvironment that slowly moves away from normal homeostasis. Even though they are stuck in the cell cycle, senescent cells keep making cytokines, chemokines, and proteolytic enzymes. Paracrine mechanisms that spread senescence to nearby cells make tissue dysfunction worse and boost inflammatory signalling. Over time, the buildup of these cells leads to a decline in both structure and function across tissues, especially in places where regeneration is limited [23].

Under normal circumstances, several regulatory systems work together to limit activation. Enzymatic breakdown of cytosolic DNA makes it harder for activating ligands to work, and autophagic mechanisms get rid of damaged organelles and stop the buildup of debris inside cells [24]. Modifications to signalling components post-translation alter the velocity of pathways and prevent excessive activity. If these regulatory mechanisms malfunction, due to factors like as ageing, oxidative stress, or genetic predisposition, signalling intensifies and inflammatory conditions persist for extended durations [25]. As nucleases exhibit diminished efficacy, cytosolic DNA accumulation occurs. When autophagy is impaired, damaged mitochondria cannot be efficiently eliminated. Alterations in the mechanisms of ubiquitination and phosphorylation complicate the cessation of normal signalling, resulting in prolonged activation beyond physiological thresholds [5]. Because these systems concurrently fail, they create settings that increase the likelihood of chronic inflammation and prolonged cellular stress responses. A comprehensive analysis of the principal molecular constituents of cGAS-STING signalling is essential for a deeper understanding of intracellular DNA sensing that triggers inflammatory and transcriptional responses, as seen in Table 1.

### 2.2. Innate Immunity in the Ovary: Cellular Interactions and Functional Implications

The immune environment in ovarian tissue is very specific and changes a lot because of structural remodelling, hormonal changes, and constant cell turnover. Folliculogenesis is a process that happens over and over again, with growth, selection, rupture, and repair. All of these steps need very precise inflammatory signalling [26]. Controlled immune activation helps the processes that lead to ovulation, such as the proteolytic remodelling of the follicular wall and changes in the blood vessels that make it easier for the oocyte to be released. After ovulation, resolution mechanisms make sure that the tissue stays intact, which stops too much damage from happening. Changes in the timing or strength of immune signalling can throw this balance off and cause physiological inflammation to become maladaptive. Local immune tone is a layer that controls the flow of endocrine signals and structural changes throughout the ovarian cycle [27].

Granulosa cells exhibit a functional profile that transcends endocrine support, integrating characteristics commonly linked to immune-responsive cells. Intracellular signalling networks let cells quickly respond to changes in metabolism and the environment, which lets them change how much cytokines they make and how they respond to stress. When inflammatory signalling activates transcriptional programs, it changes the expression of enzymes that are involved in steroid biosynthesis. This connects immune activation with hormone output. Modifications in the redox balance inside cells also change how genes are expressed and how proteins work, which affects both cell survival and differentiation. Granulosa cells are active participants in keeping local homeostasis stable even when physiological conditions change because they can process and respond to signals that indicate damage [25].

For granulosa cells and the oocyte to talk to each other, they need to be able to exchange metabolites, ions, and signalling molecules in a very coordinated way. Connexin-based gap junctions make it easier for small molecules to move directly from one cell to another. Paracrine factors control gene expression and the progress of meiosis. Alterations in how granulosa cells send signals to each other change the makeup of the secreted mediators [28]. The availability of nutrients and the signalling gradients alter around the oocyte. Alterations in how mitochondria work in granulosa cells affect the production of ATP and the balance of redox, which in turn affects the maturation of oocytes. If this finely tuned interaction is messed up, it makes it harder for spindles to form, chromosomes to line up, and the cytoplasm to mature, which lowers developmental competence. So, keeping signalling fidelity in this cellular network is important for keeping the quality of oocytes [29].

The metabolic state and organelle function of the follicular compartment are closely linked to the activity of the innate immune system. When cells are stressed, it turns on signalling pathways inside the cell that control the production of cytokines, the breakdown of proteins, and decisions about the cell’s fate. Release of inflammatory mediators creates concentration gradients in the follicular environment, which affects nearby granulosa cells, theca cells, and immune cells [9]. Recruiting and activating macrophages adds more layers of control because factors made by macrophages change angiogenesis, remodelling of the extracellular matrix, and steroidogenic activity. When somatic cells and immune components talk to each other, they form a dynamic network that can change to meet the body’s needs and respond to stress signals.

Follicular fluid is a combination of all the biochemical substances in the follicle that show the combined activity of all the cells in the follicle. Cytokines, lipid mediators, metabolites, extracellular vesicles, and nucleic acids make up the microenvironment around the oocyte [30]. Changes in cytokine profiles affect the signalling pathways that control apoptosis, proliferation, and differentiation. Extracellular vesicles carry microRNAs and proteins that change how genes are expressed in other cells, allowing signalling to go beyond direct cell-to-cell contact. Alterations in the metabolic composition, such as changes in amino acids and lipid derivatives, also affect how cells work and how well oocytes can fertilise. This level of complexity shows how important it is to think of the follicle as a single functional unit instead of a group of separate cell types [31].

Long-term imbalance in innate immune signalling changes how follicles develop and encourages pathological remodelling. Higher levels of inflammatory mediators speed up the activation of apoptotic pathways in granulosa cells and speed up the breakdown of follicles that would otherwise continue to mature. Variations signalling also affects the differentiation of theca cells and throws off the balance between androgens and oestrogens, which leads to endocrine dysregulation [32]. As more inflammatory signals build up, they make it harder for blood vessels to support follicles and deliver nutrients, which makes follicles even less likely to survive. Such forms of changes are common in conditions that cause problems with reproduction, where long-term and low-grade inflammation changes the ovarian microenvironment over time [33].

Resolution pathways are just as important for keeping the ovaries working by limiting the length and strength of immune activation. After an inflammatory stimulus, anti-inflammatory mediators, lipid-derived signalling molecules, and cellular clearance mechanisms all help bring tissues back to normal. Removing dead cells and cellular debris quickly stops inflammatory cascades from starting up again and keeps the structure of the tissue intact. When resolution processes do not work right, inflammatory signals stay around and cellular damage builds up, which makes things worse and leads to progressive dysfunction. So, the balance between activation and resolution decides whether immune signalling helps normal body functions or makes things worse.

## 3. cGAS-STING Signaling and Ovarian Ageing

### 3.1. Mitochondrial Dysfunction as a Driver of Ovarian Ageing

Ovarian function depends on sustained mitochondrial efficiency in both somatic and germ cell compartments, with energy generation intricately associated with follicular growth and oocyte maturation. As mitochondrial function declines, this equilibrium shifts early in the ageing process, despite the follicles seeming unchanged [34]. When oxidative phosphorylation becomes impaired, ATP production declines. Cells subsequently rely on alternative metabolic pathways that are often insufficient to meet their energy demands. It becomes more difficult for the follicle to coordinate its development, differentiation, and biosynthetic activity as a result of these changes in metabolism, which gradually diminishes its capacity to function in an expected manner [35].

Alterations in mitochondrial structure are an early and significant indicator of this deterioration. Electron leakage is exacerbated and the efficacy of electron flow is reduced as a result of the disorganisation of cristae architecture, which impedes the assembly of the electron transport chain. The accumulation of partly reduced oxygen species elevates oxidative stress inside the cell, impacting both mitochondrial and non-mitochondrial targets. Lipid peroxidation alters membrane fluidity and permeability, whereas the oxidation of respiratory chain proteins further diminishes their catalytic activity. The cumulative consequences induce a condition of persistent metabolic stress that becomes more difficult to reverse over time [36].

A mismatch between energy generation and consumption renders cells more susceptible. Granulosa cells, which facilitate oocyte development by providing continuous metabolic support, must meticulously regulate glycolysis, oxidative phosphorylation, and substrate transport [37]. Disruption of these metabolic pathways alters the availability of essential substrates such as pyruvate and NADH, consequently impairing energy transfer from granulosa cells to the oocyte. Oocytes possess restricted glycolytic capability and rely on the surrounding cumulus and granulosa cells for metabolic assistance during meiotic and cytoplasmic development. Reduced metabolic coupling impedes meiosis and cytoplasmic maturation, hence adversely affecting developmental competence. Even though oocyte metabolism is somewhat independent, it depends heavily on nearby somatic cells, making it especially vulnerable to interference [38].

Beyond energy metabolism, intracellular signalling mechanisms and mitochondrial dysfunction interact. Alterations in mitochondrial function influence calcium management, apoptotic signalling, and the interaction of mitochondria with other organelles, including the endoplasmic reticulum. Disruption of calcium homeostasis alters enzymatic activity and intercellular communication, hence destabilising cellular function [39].

### 3.2. Mitochondrial Instability and Intracellular DNA Mislocalization

Mitochondria often do not degrade simultaneously. Structural instability often occurs gradually, first with minor alterations in membrane composition, protein structure, and intracellular arrangement. Early alterations do not immediately impact cell survival. But they complicate the organelle’s capacity to maintain stringent regulation of its contents. As this regulation diminishes, the internal components shift more freely, particularly when the body requires substantial energy over an extended period. Chronic exposure to stress results in cumulative damage that alters mitochondrial function from adaptive to unstable [40]. 

Mitochondrial DNA is organized within nucleoid structures that are stabilized by proteins regulating mtDNA packaging, replication, and transcription, particularly mitochondrial transcription factor A (TFAM). The stability of this configuration relies on the integrity of the proteins and the biochemical conditions present in the vicinity [41]. Oxidative stress alters the structure of DNA by modifying its bases and severing strands. Because of this, it is less likely to attach to proteins that stabilise. Reduction or dysfunction of TFAM destabilizes nucleoid organization, resulting in impaired mtDNA compaction and increased susceptibility to fragmentation and cytoplasmic leakage. Increased fragmentation occurs, resulting in smaller DNA fragments being more prone to mobility. As the nucleoid deteriorates with time, mitochondrial DNA transitions from a carefully regulated genetic entity to a more unstable component of the organelle [42].

The behaviour of the membrane is crucial in determining whether these components remain confined. Alterations in lipid composition resulting from oxidative damage and inadequate lipid metabolism render membranes less stable and more susceptible to changes in permeability. Channels regulating metabolite exchange become dysregulated, remaining open for extended periods or responding anomalously to cellular signals [43]. Stress increases the frequency of pore formation, particularly in mitochondria with compromised membrane potential. Fragmented mitochondrial networks exacerbate this vulnerability, since smaller organelles possess less structural integrity. Under these circumstances, the discharge of internal material is more probable, maybe occurring several times rather than a singular instance [44].

As individuals age and experience significant stress, the cellular mechanisms responsible for maintaining cellular cleanliness become less effective. The mechanisms that degrade nucleic acids become less efficient, allowing DNA fragments that have translocated to different locations to remain in the cytoplasm for an extended duration. Autophagic processes that typically eliminate damaged mitochondria are less effective, resulting in the accumulation of malfunctioning organelles that continuously release material. Rather than repairing damage, cells perpetuate it, allowing minor issues to escalate into more significant ones [5].

Altering the molecules in mitochondrial DNA modifies its behaviour after it exits its initial compartment. Oxidative damages alter structural stability and reduce the susceptibility of DNA to enzymatic degradation. Impaired fragments often exhibit increased durability and adopt configurations that facilitate their interaction with intracellular proteins [45]. The dimensions, sequence exposure, and oxidation state of these fragments influence their cytoplasmic persistence and their impact on adjacent signalling networks. Thus, stability external to the mitochondrion is a crucial determinant in assessing the interactions among biological entities.

The cellular response is influenced by the frequency and duration of release events. Occasional leaks may be effectively managed, particularly in cells equipped with functional repair and clearing systems. However, persistent release continually induces complications inside cells. Over time, displaced DNA integrates into the stable intracellular milieu rather than remaining an anomaly. A shift like this heralds the impending dormant condition of cellular maintenance mechanisms and reveals their deeper failure [46].

Such alterations are particularly significant in the ovarian milieu due to the elevated metabolic demands of follicular cells. Granulosa cells with prolonged mitochondrial instability begin to lose their organisational capacity inside the cell, adversely impacting their signalling and metabolic coordination abilities. The accumulation of DNA fragments indicates that the cell is under significant stress, rendering the repair mechanisms ineffective in restoring equilibrium. Alterations in one region of the follicle may influence adjacent cells by modifying the surrounding biochemical milieu. This indicates that functional alterations extend beyond a single cell. The progressive proliferation of these issues destabilises follicular homeostasis and accelerates ovarian ageing.

### 3.3. Granulosa Cell Senescence and Secretory Reprogramming

As individuals age, granulosa cells do not only cease functioning; their cellular identity evolves with time. The capacity for growth diminishes, although metabolic activity remains high, resulting in a disparity between energy use and functional output. The course of the cell cycle decelerates and ultimately ceases. Long-term stress inside the cell is the cause of this; it does not occur suddenly. Despite this cessation, cells remain active and begin to adopt a phenotype that prioritises signalling above supporting follicular growth [47].

Simultaneously, metabolic behaviour undergoes alterations. The body exhibits diminished glucose handling, altered lipid metabolism, and a deceleration in mitochondrial turnover. The energy generated is insufficient to sustain coordinated follicular development, resulting in diminished cellular process coordination. Granulosa cells cease to assist egg maturation by rigorously regulating their metabolism. Instead, they start operations in a less regulated manner, characterised by fluctuations in substrate availability and redox equilibrium. This kind of instability directly impacts the metabolic milieu around the oocyte [48].

Significant alterations have occurred in the process of protein synthesis. Alterations in the activity of enzymes involved in steroid production result in fluctuations of hormone levels in the region. The synthesis of signalling molecules is less rigorously regulated, resulting in the release of more cytokines and other mediators that influence adjacent cells [49]. Communication transitions from precise paracrine signalling to a less coordinated and more diffuse mode of contact. The signalling environment around adjacent granulosa cells is distinct, potentially altering their behaviour and exacerbating local issues. Components of the extracellular matrix do not contribute to structural stability. They become more disordered. Alterations in the matrix composition modify the mechanical characteristics of the follicle, hindering cellular interactions. With the passage of time, the support structures deteriorate, and the coordination among cellular compartments declines [50].

Modifications in direct contact channels complicate intercellular communication. Gap junctions exhibit reduced functionality, impeding the transfer of metabolites and signalling chemicals across cells. When granulosa cells and the oocyte exhibit inadequate connectivity, it impedes the transport of essential substrates required for the progression of meiosis [51]. Minor issues with this communication axis may significantly impact oocyte maturation, which relies on consistent and precisely regulated assistance from adjacent cells. This altered phenotype gradually affects the entire follicular environment. Signals sent by afflicted cells influence adjacent populations, prompting alterations in their metabolic activity and signalling behaviour similarly. Gradually, a larger portion of the follicular environment becomes more inefficient. Functional heterogeneity increases, as some cells remain partly active while others become completely senescent. This results in the disruption of coordinated follicular dynamics [52].

### 3.4. Disruption of Mitochondrial Quality Control

Rather than the protracted stability of individual organelles, the integrity of mitochondria is contingent upon ongoing renewal. An equilibrium among biogenesis, fusion, fission, and the selective elimination of impaired components sustains mitochondrial function. As we age, this equilibrium deteriorates progressively, not due to a single issue, but as a result of the cumulative weakening of interrelated systems throughout time. When these pathways cease to operate in concert, organelles accumulate that are physically intact but functionally deficient, resulting in diminished cellular efficiency [53].

Mitophagy, the selective elimination of impaired mitochondria, is essential for maintaining organelle quality. Under typical circumstances, mitochondria with compromised membrane potential are identified and eliminated. As humans age, this process decelerates, allowing defective mitochondria to persist despite their inability to perform their standard bioenergetic functions [53]. Reduced turnover results in the accumulation of organelles that continuously generate reactive substances, hence increasing cellular stress. Failure to eliminate these mitochondria results in a prolonged metabolic disruption that impacts several cellular functions. Alterations in mitochondrial morphology also influence their functionality [54].

Over time, the collaboration between mitochondrial dynamics and degradation pathways becomes more challenging. Fragmented mitochondria are not always eliminated, resulting in the persistence of damaged ones within the cellular population. Simultaneously, diminished fusion complicates the resolution of issues in certain domains. This combination creates a heterogeneous mitochondrial network in which both functioning and defective organelles coexist, hence diminishing overall performance and mismatch significantly impacts cellular systems reliant on uniform energy delivery [55]. The quality control of mitochondrial proteins deteriorates, resulting in diminished functionality. Proteolytic systems that fail to maintain turnover result in the accumulation of misfolded or damaged proteins. Improper processing of mitochondrial proteins disrupts the construction of respiratory complexes, resulting in diminished efficiency of electron transport. The accumulation of fragmented components generates more reactive intermediates, exacerbating oxidative stress. Such alterations compromise the organelle’s structural integrity and metabolic efficiency [56].

As quality control mechanisms deteriorate, the communication between mitochondria and other cellular components alters. Interactions with the endoplasmic reticulum, crucial for calcium signalling and lipid exchange, diminish in precision. Altering these contact sites modifies intercellular signalling and disrupts cellular equilibrium. Alterations in calcium management significantly impact enzyme activity and may further destabilise mitochondrial function [57]. Pathways that typically modulate mitochondrial activity to satisfy cellular demands exhibit less responsiveness. Signals regulating biogenesis inadequately compensate for the loss of functioning organelles, whereas degradation mechanisms fail to effectively eliminate damaged organelles. The disparity between supply and demand perpetuates metabolic inefficiency [58]. Cells partly adapt by shifting towards alternative routes; nevertheless, these modifications do not fully restore functional capability. Mitochondrial quality control issues in the ovaries directly impact follicular health. Granulosa cells rely on efficient mitochondrial turnover to fulfil the metabolic demands of oocyte maintenance. The accumulation of defective mitochondria alters intracellular circumstances, complicating the maintenance of a stable microenvironment. The consequences extend to the oocyte, where insufficient metabolic support impedes maturation and diminishes developmental capacity. Consequently, a significant aspect of ovarian ageing is the increasing deterioration of mitochondrial maintenance systems, which makes conception more difficult [59].

### 3.5. Impact on Oocyte Competence

An oocyte’s quality is contingent upon its own characteristics, as well as the cellular environment in which it is situated. Follicular maturation requires precise coordination of metabolic support, redox equilibrium, and intracellular structure for developmental competence. Minor alterations in any of these parameters may significantly impact oocyte development, since the systems governing its development are highly regulated and provide minimal margin for mistake. As the supporting environment deteriorates, the likelihood of fertilisation and embryo development diminishes [60].

This procedure is significantly reliant on energy supply. Adequate ATP is essential for maintaining meiotic development, spindle assembly, and cytoskeletal architecture. Localised impairments impacting critical stages of chromosomal segregation result from diminished energy transfer from adjacent cells [61]. Minor fluctuations in energy balance might render spindles less stable, increasing the likelihood of misalignment or improper separation. Such alterations are intricately associated with increased incidences of aneuploidy, a characteristic hallmark of ageing oocytes. Cytoplasmic maturation is a crucial determinant of developmental efficacy. During follicular development, proteins, mRNA, and organelles accumulate in the egg, preparing it for first embryonic divisions. Alterations in metabolic circumstances influence the synthesis and storage of these components, potentially resulting in inadequate or imbalanced cytoplasmic preparation. The distribution of mitochondria inside the oocyte becomes more heterogeneous, complicating energy acquisition throughout the first stages of development. Consequently, embryos derived from such oocytes often exhibit slower development and diminished implantation potential [62].

Redox equilibrium within the oocyte is intricately linked to its capacity for development. Reactive oxygen species must be maintained at certain levels for signalling; nevertheless, excessive amounts may disrupt protein functionality and harm cellular structures. Oxidative alterations affect microtubule dynamics, impeding spindle formation and chromosomal alignment [63]. The integrity of DNA may be disrupted, increasing the likelihood of fragmentation and diminishing the embryo’s survival prospects. Maintaining a steady redox environment is crucial for preserving oocyte quality. For these circumstances to remain unchanged, communication between the oocyte and the surrounding granulosa cells is essential. Metabolites and signalling molecules are exchanged to ensure coordinated development. When this connection is disrupted, nuclear and cytoplasmic processes cease to function in concert, a phenomenon referred to as asynchronous maturation. This kind of asynchrony diminishes the likelihood of fertilisation occurring and may also impede the growth of the embryo [64].

The ramifications of these alterations extend beyond fertilisation and influence early development. Embryos derived from compromised oocytes often exhibit delayed cleavage, irregular morphology, and diminished implantation potential. Defects arising during the oocyte stage persist through subsequent developmental phases, highlighting the importance of initial cellular circumstances [60]. The quality of the oocyte is a crucial determinant of overall reproductive efficacy. Such innovations are particularly significant in the realm of assisted reproduction. Variations in fertilisation rates, embryo quality, and clinical outcomes are attributable to disparities in oocyte competence. Understanding the factors that induce these changes enables us to make informed decisions and optimise treatment outcomes. Concentrating on the biological mechanisms influencing oocyte quality may enhance reproductive success, particularly in individuals experiencing age-related fertility decrease.

### 3.6. Follicular Atresia and Loss of Functional Reserve

Follicular atresia is a fundamental mechanism that maintains ovarian equilibrium between development and apoptosis; however it manifests differently as an individual ages. Selection pressure that previously facilitated healthy follicle development gradually diminishes, resulting in increased and earlier degeneration during folliculogenesis. Elimination no longer functions as a quality-control mechanism. It signifies that follicles are incapable of managing accumulating cellular stress. A gradual deterioration in resilience shifts the equilibrium towards loss rather than development, diminishing the likelihood that follicles will reach a stage conducive to ovulation [65].

Granulosa cell survival is crucial in defining the follicle’s destiny since the follicle’s form and function are directly impacted by the health of this cell population. A reduced capacity to sustain intracellular stability alters the balance between pro-survival and pro-apoptotic signalling pathways [66]. Stress-responsive pathways gain significance, whilst anti-apoptotic mechanisms diminish in efficacy. This increases the likelihood of programmed cell death occurring. As intercellular cohesiveness gradually diminishes, the organization of the granulosa layers disintegrates, resulting in the failure of the support structures essential for ongoing development. Once the structure is compromised, it is improbable that it will regenerate, and the follicle will persist in deteriorating until it ceases to function [67].

Over time, the signalling networks regulating follicle development become less precise. The response to gonadotropins diminishes, not just due to the absence of receptors, but because the intracellular link between receptor activation and subsequent pathways is disrupted. Suboptimal signal transduction adversely affects growth, differentiation, and metabolic adaptability. Follicles that typically respond to hormonal stimulation fail to mature adequately and instead enter a condition of stalled development. The accumulation of several partly formed follicles increases the likelihood of degenerative processes over productive development [68].

Vascular support is a crucial element influencing follicular health. Proper perfusion ensures the delivery of oxygen, nutrients, and hormones essential for sustained development. Age-related alterations in the microvascular architecture diminish capillary density and impede the regulation of blood flow inside ovarian tissue [69]. In the absence of sufficient oxygen, cells must depend on less efficient metabolic pathways, exacerbating intracellular stress. Inadequate nutrition supply further impairs biosynthetic activities, reducing the ability of granulosa and theca cells to maintain coordinated activity. The synergistic effects of hypoxia and nutritional deficiency provide circumstances conducive to the initiation of degenerative processes. Alterations in the extracellular matrix also influence follicular function. The configuration and composition of the matrix components influence both signal transduction and mechanical integrity [70]. Changes in collagen deposition, proteoglycan distribution, and the activity of matrix-degrading enzymes modify the physical characteristics of the follicle. Heightened stiffness and diminished flexibility obstruct expansion throughout development stages, whilst altered matrix signalling affects cell adhesion and communication. The disruption of these structural cues impedes the integration of mechanical and biochemical signals necessary for proper follicular growth [71].

As follicles age, intercellular communication deteriorates in coordination. Granulosa cells, theca cells, and the oocyte interact by direct contact and diffusible substances. When signalling pathways lose synchronisation, metabolic cooperation diminishes, disrupting the timing of developmental processes [72]. The follicle operates as a composite structure; its many components react differently to internal and external stimuli. This kind of disorganisation diminishes the likelihood of ovulation occurring. Energetic constraints further hinder follicular vitality in ageing settings. An elevation in metabolic demand coupled with a reduction in energy production efficiency results in a condition of chronic imbalance. Cells operate nearer to their functional thresholds, indicating a diminished capacity to accommodate fluctuations in energy supply. Over time, stress accumulates and diminishes adaptive responses, rendering follicles more susceptible to both external and internal adversities. The failure to maintain metabolic balance is a significant factor driving deterioration [73].

The functional follicular pool diminishes throughout time due to cumulative changes in structure, metabolism, and signalling. Follicles that remain typically exhibit indications of diminished viability, although being advanced in their development. A decline in both quantity and quality is attributable to accelerated atresia and diminished maturation capability [74]. A reduction in functional reserve indicates not just the depletion of follicles but also a change in the overall biological environment of the ovary. In the broader context of reproductive ageing, elevated levels of follicular atresia serve as a crucial connection between cellular malfunction and clinical deterioration. Insufficient healthy follicles diminish the likelihood of fertilization [32]. The persistence of dysfunctional follicles further diminishes overall efficiency. The self-perpetuating cycle that hastens reproductive decline is established by the interplay between degenerative processes and hindered maturation. Comprehending the temporal alterations of these processes may explain age-related infertility and guide us toward potential methods for preserving functional reserve.

### 3.7. Systemic Extension Beyond the Ovary

Reproductive ageing occurs simultaneously across all components of the reproductive system, not just inside the ovaries. Functional integrity depends on the synchronisation of ovarian function, endometrial responsiveness, vascular dynamics, and immunological control. Gradually diminishing coordination among these systems alters the timing and efficacy of critical reproductive events, despite the individual components being physically sound [75]. Disruption of this synchrony reduces the probability of successful implantation and the maintenance of pregnancy. The endometrium’s function alters in manners that influence its capacity to facilitate embryo implantation. Other cellular composition transitions to a less responsive condition, impacting the integrity of the epithelium, the differentiation of the stroma, and the local signalling networks [76]. A reduced ability for proper decidual transformation affects the uterine lining’s readiness for embryo implantation. Alterations in gene expression patterns influence the concentrations of adhesion molecules and growth factors essential for successful implantation. Thus, embryos with adequate developmental potential may encounter adverse circumstances after their transfer to the uterine environment [77].

As individuals age, their hormonal responsiveness diminishes. Signalling mechanisms that translate endocrine cues into synchronised cellular responses diminish in precision, leading to asynchronous reactions in target tissues. Alterations in oestrogen and progesterone signalling impact both the ovaries and the endometrium, complicating the synchronisation of follicular development and uterine preparation [78]. Modifications in receptor activation and the efficacy of downstream signalling result in less reliable reproductive results. This diversity adds an additional layer of complexity to comprehending age-related infertility. Vascular function is crucial for maintaining reproductive health and undergoes alterations with ageing. Improvements in endothelial integrity and microvascular regulation affect blood flow inside reproductive tissues [79]. Reduced perfusion impedes the delivery of oxygen and nutrients to the cells, while adverse vascular remodelling hinders the endometrium’s preparation for implantation. Changes in angiogenic signalling render the environment more inhospitable for early embryonic development. Proper vascular adaptation is essential for follicular maturation and implantation, making its decrease a critical aspect of reproductive ageing [80].

Over time, the uterine immune system’s functioning also varies. Achieving equilibrium between tolerance and activation is more difficult, affecting the ability to promote implantation while reducing excessive inflammation. Modifications in cytokine profiles and immune cell populations influence local signalling networks that regulate tissue morphogenesis and the embryo’s interaction with the mother. Minor alterations in the immune system might disrupt the fragile equilibrium essential for successful implantation, particularly during the first phases of pregnancy [81].

The connection between ovarian and uterine compartments is more evident when assessing overall reproductive results. Alterations in the ovary influence the quality of the egg and early embryo, while modifications in the uterus impact the likelihood of implantation and the advancement of pregnancy. When various components fail to function cohesively, the whole system’s efficiency diminishes, notwithstanding the continued operation of some portions. A synchronised reduction across systems is therefore a crucial indicator of reproductive ageing [82].

Clinical manifestations of this systemic process include diminished implantation rates, elevated early pregnancy loss rates, and varied reactions to assisted reproductive techniques. The ovarian reserve alone cannot entirely explain discrepancies in treatment results, highlighting the impact of extragonadal variables. Being aware of this complete framework shifts focus from individual organ function to integrated system dynamics. Understanding the interplay among different compartments in the context of ageing may improve diagnostic and treatment approaches. From a translational perspective, concentrating just on a singular component of the reproductive system may be insufficient to restore complete functionality. Strategies that include both the ovarian and uterine environments, together with their molecular pathways, are more likely to provide substantial improvements [83]. Integrating metabolic, vascular, and immunological aspects into treatment strategies may enhance reproductive results, particularly for those facing age-related deterioration. Ongoing examination of these interconnected mechanisms is essential for the progress of reproductive medicine.

## 4. cGAS-STING Signaling in the Follicular Microenvironment

### 4.1. Inflammatory Signaling Within the Follicular Niche

Inflammatory activity inside the follicle adheres to a certain pattern that promotes normal development rather than inhibiting it. Precise regulated release of signalling molecules transpires throughout essential stages of folliculogenesis, enabling the synchronising of growth, differentiation, and structural remodelling [84]. Proper modulation ensures that inflammatory signals are transient and localised, preventing unnecessary activation from spreading. Ongoing adaptation to metabolic conditions and cellular status is essential for the stability of this system, as even minor imbalances can alter the course of follicular maturation.

Granulosa cells play a crucial role in establishing this signalling environment due to their ability to rapidly modulate secretion levels. Alterations in intracellular metabolism, oxidative status, and stress exposure result in changes to the profile of released mediators. Interleukins, chemotactic factors, and growth-related chemicals are synthesised in patterns that reflect the existing cellular environment rather than preordained programming [85]. As follicles mature, their secretion characteristics alter, influencing the growth, transformation, and viability of adjacent cells. The follicle may adapt to changing requirements due to the dynamic nature of this process. Still, this also renders it more susceptible when regulatory systems fail [72].

The distribution of signalling molecules in the follicular region is contingent upon their diffusion and spatial arrangement. As substances traverse follicular fluid, they create concentration gradients that vary across various regions of the follicle. Cells proximal to the secretion source may have more exposure or prolonged contact compared to those situated distally. Alterations in fluid composition, viscosity, and the arrangement of the extracellular matrix may influence the distance and velocity of signal propagation. Spatial variation in exposure results in diversity in cellular responses, particularly as signalling becomes less controlled [86].

Prolonged elevations in inflammatory mediators alter follicular function. Signalling that typically aids in coordination begins to induce instability when prolonged excessively. Cellular responses exhibit less coordination, resulting in increased disparities in activation states among adjacent cells [87]. Gene expression patterns shift towards stress-related programs, and metabolic pathways alter in ways that do not consistently facilitate healthy growth. The incremental buildup of these alterations diminishes communication efficacy and complicates the collaboration of follicular compartments [88,89].

The incorporation of immune cells into the follicular niche represents an alternative method of regulation. Macrophages and other immune cells engage with follicular cells via direct contact and soluble mediators. Their action facilitates angiogenesis, extracellular matrix remodelling, and the elimination of cellular waste. When circumstances are optimal, these interactions promote follicular growth. Prolonged stress may amplify immune-derived signals, exacerbating local disruptions and creating a pro-inflammatory environment that affects cellular behaviour inside the follicle.

Temporal synchronisation is crucial for ensuring operational efficiency. To facilitate the successive phases of follicular growth, signalling events must occur within certain time windows. Premature activation or postponed resolution might impede advancement, resulting in the overlap of phases that need to remain distinct. When timing is misaligned, it complicates the interplay of proliferation, differentiation, and maturation, hence impeding the whole developmental process. The timing of inflammatory signalling is just as crucial as its strength in determining outcomes [90].

### 4.2. Follicular Fluid as a Functional Readout

Follicular fluid precisely reflects the current conditions inside the follicle. The substance’s composition indicates the efficiency of the granulosa and theca cells, the coordination of metabolic processes, and the suitability of the surrounding region for oocyte development. Alterations often occur here prior to becoming seen at the morphological level, making it a very sensitive indicator of follicular health [91].

It is crucial for this fluid to maintain a balanced metabolism. The concentrations of amino acids, lipids, and energy substrates fluctuate based on cellular activity levels. When everything is well-regulated, these components remain within a range that facilitates sustained energy production and biosynthesis. When equilibrium is disrupted, predicting substrate availability becomes more difficult, and cells operate under suboptimal circumstances. Granulosa cells may fail to adequately nourish the oocyte during maturation in response to even minor alterations. Lipids enhance the complexity of this ecosystem. They influence membrane structure, receptor functionality, and intercellular communication. Alterations in lipid profiles may indicate more significant metabolic issues, particularly when mitochondrial function is impaired. Certain lipid types may accumulate, whilst others may degrade [92].

Cytokines and growth-related mediators provide more insight into the local regulatory milieu. Their concentrations indicate cellular responses to internal changes, including stress and energy requirements. When equilibrium is achieved, signalling remains regulated and facilitates coordinated development. If this equilibrium shifts, signalling accuracy diminishes [93]. Cells begin to receive mixed or prolonged messages, which may disrupt coordination within the follicle and impair overall function. Extracellular vesicles facilitate intercellular communication via mechanisms beyond mere physical contact. They transport proteins, microRNAs, and other substances that may alter gene expression in adjacent cells. The contents vary according to the condition of the cells that secrete them. This mechanism facilitates synchronisation inside a well-functioning follicle. Under stress, it may emit signals indicative of malfunction, hence intensifying within the follicular environment [94].

From a therapeutic perspective, follicular fluid provides essential information that cannot be obtained from systemic assessments. Differences across follicles during a single cycle highlight the impact of local circumstances on oocyte quality. Certain patterns are associated with increased rates of fertilisation and embryo development, whilst others correlate with worse outcomes. Context is essential for comprehension, since no single indicator conveys the whole narrative. Nevertheless, aggregated profiles may provide valuable insights.

### 4.3. Intercellular Communication and Extracellular Vesicles

Communication inside the follicle goes much beyond direct connections among neighbouring cells. Gap junctions facilitate the rapid transfer of tiny molecules across cells. But significant coordination relies on signals sent by the follicular fluid. Extracellular vesicles are crucial to this process since they transport information that may influence distant cells, modulating their behaviour in a nuanced and regulated manner [95].

Granulosa cells consistently secrete vesicles into their surrounding environment. Their contents indicate the current state of the cells. MicroRNAs, proteins, and lipids are assembled and then internalised by various cells inside the follicle, including the oocyte. In this manner, cells may communicate their metabolic status, stress levels, and general activity to one another. This enables the follicle to function as a cohesive unit rather than a collection of disparate components. MicroRNAs are crucial since they can precisely regulate gene expression. Minor fluctuations in their concentrations might alter whole pathways, thus affecting cellular growth, differentiation, or stress responses. This maintains alignment inside the follicle under steady conditions. MicroRNA levels fluctuate in response to changing situations, often indicating that the body is progressing towards adaptability rather than ideal functionality. Such modification may influence the efficacy of the follicle in facilitating oocyte maturation [96].

Proteins inside vesicles facilitate this communication. Enzymes and signalling molecules may be directly transferred into recipient cells, altering intracellular pathways without necessitating the synthesis of new genes. Vesicles may alter membrane characteristics and signal reception and processing by transporting lipids and are capable of altering numerous aspects of cell function simultaneously and collaborate to facilitate this process [97]. This mechanism acquires an additional dimension upon interaction with the oocyte. Vesicles may transmit signals that alter cytoplasmic organization, mitochondrial function, and the cell’s preparedness for fertilisation. The oocyte is very sensitive to its environment, so that even indirect influences may have significant repercussions. Differential vesicle makeup may explain the erratic behaviour of oocytes from the same cycle [78].

Vesicle exchange also influences the communication between granulosa cells. Cells in such circumstances often assist one another by emitting signals and ensuring consistency in a well-functioning follicle. However, in the presence of stress, altered signals may proliferate and exacerbate the imbalance throughout the follicle. The generation of vesicles is contingent upon cellular energy and membrane dynamics. When metabolism functions well, vesicle generation and cargo selection are effectively regulated. The accuracy of this mechanism diminishes when the energy balance is disrupted. Cells may exocytose vesicles containing various substances, and the mechanisms by which receiving cells internalise them may also vary. This results in communication being less precise and more inconsistent.

### 4.4. Clinical Implications for Oocyte Quality and IVF Outcomes

Discussions on results in assisted reproduction often emphasise numerical data. Despite this, the biological processes occurring inside each follicle are as significant. The quality of an oocyte is contingent upon the synchronisation of many intracellular processes throughout maturation. Despite favourable shape, issues with metabolism or signalling might adversely affect developmental potential. Variations in the local microenvironment of oocytes from the same cycle often induce discrepancies, in addition to systemic variables [98].

Energy management is critically significant. The efficiency of mitochondrial function in granulosa cells influences the consistency of metabolic substrate delivery to the oocyte. When this support becomes unstable, the availability of ATP during critical phases, such as spindle formation and chromosomal alignment, may fluctuate. Even minor variations at this level may alter the organization of microtubules and increase the likelihood of segregation errors. This clarifies the causes of oocyte failure, despite their morphological normality at the moment of extraction [59].

Signalling routes inside the follicle gradually affect oocyte competency. Changes in stress-related signalling and inflammatory tone influence the behaviour and interaction of granulosa cells with the oocyte. The prolonged stimulation of these pathways does not always result in evident detriment. Nonetheless, it may alter cellular priorities, shifting their emphasis from optimal development to mere survival. As a result, the oocyte may complete meiosis without having the cytoplasm adequately prepared for optimal embryo development [99].

Alterations in mitochondrial stability and turnover influence energy generation and intracellular signalling. Uneven distribution of mitochondrial material in the oocyte may result in an inconsistent energy supply during the first divisions of the embryo. At fertilisation, these effects are rarely noticeable [59]. However, they may be observed in the future as delayed cleavage or reduced developmental progress.

Communication via extracellular vesicles adds a sometimes overlooked feature. Signals sent in this manner may alter gene expression without direct interaction, impacting both granulosa cells and the oocyte. When vesicular contents signify a stressed or dysregulated state, this information might propagate throughout the follicle, simultaneously affecting several cells. Variations in vesicular signalling may explain why oocytes within the same cohort often have distinct appearances [100].

Follicular fluid provides insight into localised biology in a way that systemic indicators cannot. The composition illustrates the ongoing interconnections between metabolism, signalling, and cellular stress. Patterns involving mixtures of metabolites, cytokines, and vesicle-derived chemicals correlate with fertilisation rates and embryo quality. A thorough study of these profiles, rather than reliance on individual markers, may provide a greater knowledge of which oocytes are more likely to succeed [101].

To get improved outcomes, we may need to concentrate more on the underlying biology rather than only on stimulation tactics. Strategies that improve mitochondrial function, reduce cellular stress, or regulate local signalling may profoundly influence oocyte competence. There is a growing interest in metabolic regulation and tailored therapies, particularly among patients facing recurrent implantation failure or inadequate embryo growth. Table 2 depicts the molecular determinants of oocyte competence.

## 5. cGAS-STING Signaling in Reproductive Pathology

### Polycystic Ovary Syndrome and Metabolic-Inflammatory Crosstalk

Reproductive problems seldom arise from a solitary anomaly. A more precise representation involves a progressive divergence from intracellular equilibrium, during which metabolic stress, organelle instability, and altered signalling begin to overlap and exacerbate one another. Ovarian and endometrial cells operate inside systems that need meticulous coordination of energy generation, chromatin arrangement, and intercellular communication [102]. Cells begin to operate under an altered hierarchy of priorities as soon as this coordination begins to deteriorate, even little. Early changes often remain undetected and lack evident structural consequences. They influence mitochondrial efficiency and redox equilibrium. Over time, compensatory reflexes dominate, establishing a new baseline that enables survival but hinders optimal performance [103]. Signalling pathways intended to be transient remain active longer than anticipated, gradually altering the functionality of transcriptional programs. Functional degradation occurs, first at the molecular level, then presenting as impaired follicular growth, reduced endometrial receptivity, and finally infertility.

Polycystic ovarian syndrome illustrates the internal processes of this transformation inside the follicle. Prolonged exposure to elevated testosterone levels and insulin signalling dysfunction alters the energy utilisation of granulosa cells [104]. Glucose management becomes less effective, lipid metabolism undergoes alterations, and mitochondrial respiration undergoes a loss of regulation. The electron transport across the respiratory chain becomes less stable, resulting in leakage and an increased formation of reactive oxygen species. Over time, oxidative stress alters the proteins, lipids, and general architecture of the organelle [105]. The membrane potential exhibits instability, ATP generation fluctuates, and calcium regulation is compromised. Subsequently, structural alterations occur, including the disorganisation of cristae and the heightened permeability of mitochondrial membranes. Both of these factors compromise compartmental integrity.

The loss of fundamental integrity has repercussions that extend beyond energy production. During oxidative stress, mitochondrial DNA, typically safeguarded by structured nucleoids, becomes more vulnerable. As damage accumulates, the mechanisms that maintain and repair mitochondrial DNA become more ineffective. The pace of damage does not consistently correspond with the rate of clearance mechanisms, such as mitophagy [106]. Consequently, pieces last longer than anticipated and may appear in locations where they are often absent. Granulosa cells alter their intracellular signalling mechanisms in response. Pathways associated with stress and inflammation increase, whilst those facilitating differentiation and steroidogenesis diminish in stability. Aromatase activity becomes erratic, oestrogen synthesis is less stringently controlled, and the body’s responsiveness to gonadotropins diminishes. Follicles continue to develop, although they lose their synchronisation, resulting in the cessation of the disease [107].

Endometriosis occurs within a specific anatomical framework, despite many underlying factors being similar. Ectopic endometrial cells inhabit an environment characterised by unstable oxygen supply and elevated oxidative stress. Mitochondria in these cells are consistently under stress, resulting in diminished efficacy of oxidative phosphorylation and increased significance of glycolysis [108]. This alteration in metabolism aids cellular survival, although it also leads to the accumulation of intermediates that influence intercellular communication. Reactive oxygen species accumulate, causing damage to proteins, lipids, and nucleic acids. Damage impacts both nuclear and mitochondrial DNA, complicating the efficacy of repair mechanisms [109].

Repair mechanisms do not consistently operate with sufficient speed. Degraded DNA fragments accumulate and alter cellular regulatory mechanisms. Stromal cells in endometriotic lesions gradually enhance their invasive capabilities and exhibit reduced apoptosis [110]. Autophagy-related pathways become more active, aiding cellular survival under adverse conditions, while concurrently facilitating the persistence of the lesion. Simultaneously, engagement with immune cells perpetuates a loop that is difficult to disrupt. Macrophages and other immune cells secrete cytokines that amplify inflammatory signals and promote tissue remodelling rather than resolution. The consequences extend beyond the lesion itself. Signals sent into the external environment influence ovarian function by altering the circumstances inside the follicle and impacting granulosa cells [111].

Endometrial dysfunction introduces an additional complexity, particularly with implantation. Successful implantation necessitates the synchronisation of the epithelial and stromal compartments at the appropriate temporal juncture. Cells must differentiate in a regulated manner, maintain their integrity, and appropriately react to hormonal cues [112]. This coordination begins to deteriorate under prolonged cellular stress. The nucleus’s structure deteriorates, and the mechanisms that typically safeguard DNA and maintain its proper localisation become less efficient. Fragments that would typically be confined or eliminated may persist and disrupt signalling [113].

Alterations at this level influence gene expression; however their effects may not be immediately observable. The timing and consistency of the processes associated with decidualisation, adhesion, and vascular adaptation are disrupted. Certain signals arrive prematurely, whilst others arrive tardily, complicating their collaborative efficacy [114]. There exists increased geographical diversity, with distinct regions of the endometrium exhibiting differential responses. Despite an embryo’s viability, implantation may not occur due to a misaligned environment. The issue lies not in the absence of requisite signals, but in their lack of coordination. All of these processes are influenced by hormonal control. Oestrogen influences mitochondrial function and redox equilibrium, while progesterone facilitates cellular differentiation and immunological tolerance. Hormonal signals maintain organization under steady conditions. Hormonal intervention may be ineffective if intracellular signalling is already disrupted. Responses vary based on receptor sensitivity and the efficacy of downstream signalling mechanisms. Certain cells react appropriately, while others do not, exacerbating coordination within the tissue [115].

A recurring trend begins to emerge while examining these events. Mitochondrial malfunction, altered intracellular DNA regulation, and persistent activation of stress-related signalling pathways often coexist. This pattern modifies cellular function after it is formed. Communication becomes less clear, compartmentalisation diminishes, and signalling persists longer than necessary [116]. Moreover, the consequences are not restricted to a single tissue; they also influence the immune system, endometrium, and ovary. Clinically apparent structural alterations indicate a more advanced stage of a process that begins at the molecular level somewhat earlier. Reproductive disease may be seen as a gradual decline in intracellular structure rather than a sudden failure. Cells shift from orchestrating coordinated functions to managing chronic stress [117]. In clinical contexts, this alteration may not manifest immediately. However, it has a significant impact on numerous aspects of reproductive capacity. Examining these illnesses from a common molecular perspective facilitates the integration of disorders often seen as distinct. It facilitates the use of more integrated approaches for comprehending and addressing them [118]. The involvement of cGAS-STING signaling across reproductive disorders is summarized in Table 3.

## 6. Therapeutic Perspectives and Targeting cGAS-STING

Interest in targeting intracellular stress pathways has grown, since it is clear that many reproductive problems are not just hormonal or anatomical, but fundamentally linked to cellular malfunction. The processes occurring inside granulosa cells, oocytes, and endometrial tissue often dictate the result far in advance of any observable clinical manifestations [118]. Initiatives to improve fertility are increasingly focusing on the quality of the cellular environment that supports reproduction, rather than just on stimulation.

The direct manipulation of cGAS-STING communication is a very promising domain. Experimental study demonstrates that STING inhibition may reduce inflammatory signalling triggered by intracellular DNA accumulation, especially in situations marked by mitochondrial instability [120]. Reducing this background activation may assist cells in returning to a more regulated state, whereby signalling is just transient. In granulosa cells, this may indicate enhanced stability of steroidogenesis and improved responsiveness to gonadotropins. Simultaneously, total suppression would be detrimental, since transient activation of innate pathways fulfils a function in the organism. Balance is likely the primary concern, rather than the elimination of items [121].

Mitochondrial assistance remains crucial due to the frequent occurrence of issues at this level across many contexts. Improving mitochondrial function entails not only increasing energy generation but also stabilising the intracellular environment. As respiration improves and oxidative stress diminishes, the accumulation of damaged components decreases, resulting in more stable signalling. Antioxidants have been used for an extended period [122]. The outcomes have not always been favourable. The consequences are likely contingent upon the circumstances. More focused tactics, such as enhancing mitochondrial dynamics or optimising quality control mechanisms, may be more effective. Even little improvements in mitochondrial stability may profoundly affect the function of granulosa cells in aiding the oocyte [123].

Metabolic treatments provide a supplementary pragmatic strategy. In polycystic ovarian syndrome, enhancing insulin sensitivity or modifying substrate availability might indirectly reduce cellular stress. Dietary modifications or the use of metabolic agents may promote the reestablishment of a stable energy balance, hence affecting cellular regulation of oxidative stress and signalling pathways [124]. Certain individuals believe that ketone bodies may enhance mitochondrial function in certain contexts. But their involvement in human reproduction remains ambiguous. Metabolism and signalling are obviously interconnected, and enhancing one often improves the other as well [125].

The regulation of intracellular DNA is an emerging area of study. When mitochondrial components are not adequately removed, remnants persist and continue to influence signalling pathways. Facilitating mechanisms such as mitophagy or accelerating mitochondrial turnover may address this issue. This concept remains mostly in the experimental phase [126]. It aligns with the notion that it may be more advantageous to prevent harm accumulation rather than to address the subsequent impacts. Researchers are examining extracellular vesicles from a novel perspective, seeing them as both biomarkers and potential instruments. The contents of these cells indicate the health of their originating cells, making them valuable for diagnostic purposes. There is increasing interest in the potential of vesicles to transport regulatory chemicals to specific regions inside cells [127]. In principle, modifying vesicle content may influence gene expression inside the follicle. The practical application remains distant, although it illustrates the potential for altering intercellular communication rather than only observing it [78].

## 7. Discussion

**Figure 1:**
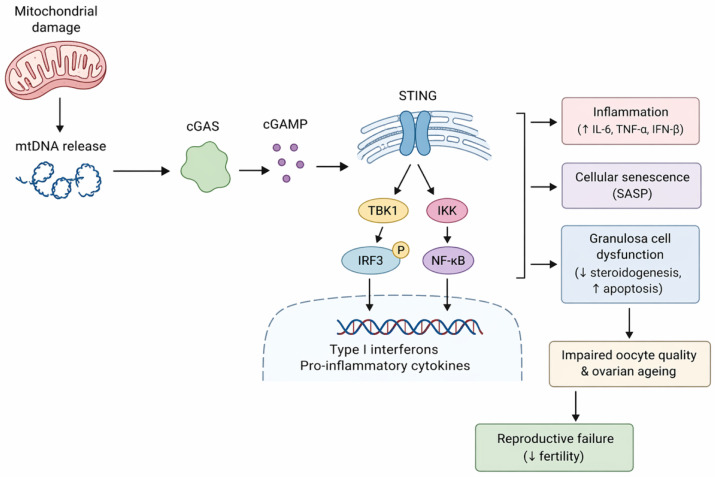
cGAS-STING signaling links mitochondrial dysfunction to inflammation, ovarian ageing, and reproductive failure.

Data from ovarian biology, the follicular milieu, and reproductive pathology converge on a singular paradigm whereby intracellular stress, mitochondrial malfunction, and aberrant DNA sensing together diminish reproductive efficacy. cGAS-STING signalling functions as a crucial integrator of these pathways, linking metabolic imbalance to inflammatory activation and cellular senescence. The alterations in granulosa cell activity, oocyte maturation, and endometrial receptivity seem to progress along the same trajectory. Constant activation of stress pathways gradually alters the priorities of cells, diverting them from coordinated growth. This approach clarifies the underlying causes for the common molecular traits seen in many clinical diseases, including polycystic ovarian syndrome, endometriosis, adenomyosis, intrauterine adhesions, and implantation failure, despite their unique phenotypic manifestations. To better combine these processes in a more integrated fashion, a simplified overview of the proposed mechanism is shown in Figure 1.

The figure depicts the stepwise changes in the follicular environment due to mitochondrial stress. Mitochondrial damage results in the release of mitochondrial DNA into the cytoplasm, where it is sensed by cGAS. This activates STING and downstream signalling through TBK1, IRF3 and NF-κB resulting in sustained inflammatory activity. This affects granulosa cell function in the long term, induces cellular senescence and disturbs the balance essential for normal oocyte development. This leads to a deterioration in the quality of oocytes and reproductive performance.

The first research that established the route offers a robust biological foundation for this hypothesis. Ishikawa et al. showed that STING serves as a crucial adaptor for type I interferon responses elicited by intracellular DNA [128]. This indicates that misplaced DNA may serve as a potent innate immune signal rather than only a consequence of damage. Sun et al. subsequently identified cGAS as the primary cytosolic DNA sensor responsible for synthesising cyclic GMP-AMP [127]. This addressed the gap in the process linking DNA recognition and STING activation. Gao et al. and Ablasser et al. enhanced this paradigm by demonstrating that cGAS synthesises a cyclic dinucleotide characterised by a distinctive structure and mixed phosphodiester linkages [129,130]. This elucidates the reasons for the robust and selective downstream activation of STING. Although these investigations did not focus on reproductive organs, they are crucial for comprehending subsequent events in the ovaries and endometrium. They demonstrate that cells possess a highly conserved signalling mechanism capable of converting DNA located outside its typical compartment into inflammation, interferon signalling, and extensive transcriptional reprogramming.

Yan et al. were the pioneers in demonstrating the functional significance of this mechanism in the ovary [111]. They demonstrated that granulosa cells consistently express cGAS, STING, and other cytosolic DNA sensors, enabling them to initiate a rapid innate immune response upon intracellular DNA stimulation. This result is crucial as it reclassifies granulosa cells from ordinary endocrine support cells to stress-responsive immunometabolic cells. Their model demonstrated that exposure of cells to viral DNA analogues induced interferon production, inflammatory cytokine release, antiviral program activation, and alterations in steroidogenic activity. This indicates that DNA sensing in granulosa cells is not physiologically neutral in practice. Upon activation, it may alter the inflammatory tone and endocrine activity inside the follicle.

Liu et al. significantly expand upon this concept by demonstrating that this mechanism may be activated in the absence of any viral DNA [107]. Their model demonstrates that toxic damage may destabilise mitochondria, induce the leakage of mitochondrial DNA, and directly activate cGAS-STING signalling in granulosa cells. This was associated with increased TNF-α production, inflammatory cell infiltration, and ovarian fibrosis. Critically, transient activation of cGAS-STING signalling may not be deleterious in physiological settings as short-term innate immune activation can aid in cellular stress adaptation and intracellular surveillance. In contrast, prolonged or aberrant activation seems to drive granulosa cells towards chronic inflammatory signalling, senescence-associated responses and progressive tissue dysfunction. Pan et al. assert that mtDNA leaking is pivotal to granulosa cell ageing, showing that Immp2l deficiency triggers senescence-associated secretory signalling via cGAS-STING activation [91]. Their subsequent research elucidates the pathway further by linking TFAM dysregulation, CyPD40, and VDAC1 to mitochondrial instability and the release of mtDNA into the cytoplasm. This delineates a more precise structural pathway via which mitochondrial failure induces inflammatory signalling. Qu et al. clarify this situation by showing that Immp2l loss compromises mitochondrial proteostasis via reduced UPRmt and inefficient mitophagy, with pathways associated with STAT1/ATF4 and HIF1α/BNIP3 playing a role in granulosa cell senescence [131]. Qu et al. and Pan et al. together indicate that the activation of cGAS-STING in ageing granulosa cells is not a unique phenomenon. Rather, it is a component of a broader failure in mitochondrial quality control, whereby oxidative stress, impaired proteostasis, faulty turnover, and mtDNA instability converge to a shared inflammatory outcome [91,131].

This extensive model is particularly relevant in metabolically dysregulated ovarian conditions. Zhao et al. demonstrate that in a letrozole-induced PCOS model, hyperandrogenism undermines mitochondrial integrity, promotes the leaking of cytochrome c and mtDNA, and activates both apoptotic pathways and cGAS-STING-NF-κB signalling in granulosa cells [105]. Their finding that ketogenic intervention and β-hydroxybutyrate may partly reverse this phenotype is very valuable, as it demonstrates that pathway activation is not just indicative of damage but can also be modulated by metabolic processes. Ovarian dysfunction associated with PCOS may indicate not just excessive hormonal activity but also a condition whereby mitochondrial stress and innate immune signalling are interconnected. Key et al. support this hypothesis by demonstrating that disruption of mitochondrial proteostasis may initiate cGAS-STING-mediated innate immunological responses in the setting of infertility [132]. Tsui et al. demonstrate that DNA repair deficiencies may partly activate the cGAS-STING system postnatally, hence enhancing its relevance to gametogenesis. These investigations reveal a distinct pattern: metabolic strain, mitochondrial instability, and genomic stress do not function as isolated insults; rather, they together impact the same intracellular alarm system [133].

A similar convergence is seen in uterine ageing and impaired implantation. Chen et al. demonstrate that the uterine tissue of aged mice accumulates cytoplasmic DNA, has elevated levels of progerin and distinct lamina-associated proteins, and activates cGAS-STING signalling concurrently with a decline in receptivity [92]. Their finding that high oestrogen might further increase progerin and cytoplasmic DNA accumulation, while STING inhibition somewhat mitigates uterine abnormalities, is very striking. This transcends the notion that uterine ageing is only attributable to hormonal or structural factors. It posits that age-related nuclear instability and DNA mislocalization facilitate the sustained activity of the innate immune system, hence directly influencing implantation-related signalling. This model well corresponds with the central notion presented in our review, whereby reproductive ageing denotes a decline in intracellular structure and a decrease in endocrine effectiveness.

Investigations into intrauterine adhesions further substantiate this assertion by demonstrating that cGAS-STING activation is associated with tissue remodelling and fibrosis, rather than only inflammation. Wu et al. demonstrate that the leakage of mitochondrial DNA may induce the transformation of endometrial epithelial cells into mesenchymal cells, and that pharmacological inhibition of STING reduces both inflammatory and fibrotic markers [88]. Their supplementary research places XBP1 at the forefront of this reaction, suggesting that stress-adaptive transcriptional mechanisms might actively promote mtDNA release and cGAS-STING-mediated fibrotic remodelling. These findings cumulatively demonstrate that DNA-sensing pathways communicate cellular damage and promote the rearrangement of endometrial tissue in ways directly linked to infertility, recurrent pregnancy loss, and implantation failure. Li et al. demonstrate that inflammatory stimulation with LPS induces mitochondrial dysfunction in endometrial stromal cells, resulting in mtDNA leakage and the production of STING-dependent cytokines [119]. Endometrial and ovarian pathologies have similar molecular patterns characterised by organelle malfunction, aberrant DNA localisation, and persistent inflammatory signalling, which together exacerbate the conditions.

Endometriosis and adenomyosis provide an additional dimension to this signalling pathway, which facilitates the persistence and detrimental transformation of lesions. Zhu et al. demonstrate that the activation of cGAS-STING enhances autophagy and facilitates the motility and invasiveness of endometrial stromal cells. Conversely, inhibiting STING reduces the incidence of ectopic lesions in vivo [134]. This discovery is particularly compelling since it links DNA sensing to both inflammation and the survival mechanism of ectopic tissue. Lin et al. assert that adenomyotic tissue exhibits elevated levels of cGAS, STING, TBK1, IFN-related mediators, and TNF-α, with these pathways being activated in the presence of dysmenorrhea and uterine enlargement [15]. Ye et al. provide a microenvironmental viewpoint, showing that ovarian endometriotic cyst fluid induces cGAS-STING-dependent macrophage inflammation and senescence, as well as mitochondrial dysfunction, and that STING suppression by H-151 may alleviate ovarian damage [123]. These results demonstrate that cGAS-STING signalling functions not just as a cellular stress response, but is integral to broader inflammatory networks including stromal cells, immune cells, and ovarian tissue. Once established, these circuits likely sustain lesions and diminish oocyte quality while disrupting follicular activity.

Evidence for the overarching reproductive importance of this route is also obtained from non-ovarian models. Jia et al. demonstrate that oxidative mitochondrial DNA may activate cGAS-STING and subsequent inflammasome signalling in testicular failure associated with varicocele [135]. Zhu et al. demonstrate that particulate matter harms mitochondria and induces cGAS-STING-dependent orchitis, which may be ameliorated by aspirin-mediated cGAS acetylation [136]. Deng et al. enhance this framework by linking heat stress in spermatogonia to cGAS-STING activation and pyroptosis, whereas Moxuan Zhao et al. illustrate that micro- and nanoplastics impair mitochondrial dynamics, provoke mtDNA translocation, and initiate cGAS-STING-mediated inflammation and pyroptosis in testicular tissue [137,138]. Although they are male models, their relevance to the present study lies in the consistency of the upstream biology. Various forms of damage, including thermal injury, environmental pollution, oxidative stress, toxic exposure, and mitochondrial fission dysregulation, contribute to mtDNA mislocalization and the activation of the cGAS-STING pathway in reproductive organs. This convergence clearly indicates that this route serves as a crucial stress-sensing hub in reproductive biology, rather than being an observation limited to certain settings.

The biology of ageing provides an additional dimension of comprehension that integrates all of these data. Glück et al. established that cytoplasmic chromatin fragments from senescent cells activate cGAS and enhance the senescence-associated secretory phenotype via STING [139]. Yang et al. further demonstrated that cGAS is essential for the initiation of cellular senescence, establishing DNA sensing as fundamental to the process by which injured cells transition into chronic inflammatory states [140]. Sladitschek-Martens et al. demonstrated that YAP/TAZ activity inhibits cGAS-STING signalling by maintaining the integrity of the nuclear envelope, hence safeguarding against tissue ageing [141]. These discoveries are crucial for reproductive medicine since they demonstrate the interconnection between structural deterioration, DNA mislocalization, persistent inflammation, and tissue-level ageing. In ovarian and endometrial systems, where success depends on exact timing, coordination, and cellular integrity, the sustained activation of such a route is expected to have notably severe consequences.

This review consolidates research that endorses a cohesive and physiologically reasonable concept whereby cGAS-STING signalling functions as a molecular link among ovarian ageing, inflammation, and infertility. Numerous upstream factors exist, including metabolic stress, mitochondrial malfunction, endocrine imbalance, environmental exposure, defective DNA repair, and age-related nuclear instability [142]. Nevertheless, the downstream reasoning remains quite consistent. DNA appears in inappropriate locations, intracellular sensing is activated, inflammatory and interferon-related signalling is persistent, and cells shift their focus from coordinated reproductive support to stress adaptation and survival. This indicates that granulosa cells in the ovary exhibit dysfunction, undergo ageing, alter steroidogenesis, provide inadequate support to oocytes, and experience follicular loss [143]. It results in inadequate receptivity, fibrosis, inflammatory remodelling, and suboptimal embryo-maternal synchronisation in the endometrium. At the systems level, it elucidates why illnesses that present differently in clinical settings often have analogous biological characteristics and often coincide with reproduction issues.

A final note from this synthesis is that cGAS-STING signalling should not be seen only as an inflammatory outcome. Evidence suggests it plays a more upstream role, synthesising mitochondrial quality, intracellular DNA regulation, endocrine influences, and tissue ageing into a unified functional response. This is particularly intriguing for concepts, since it provides a means to integrate many domains of reproductive illness that are often examined in isolation [144]. Concurrently, current research mostly centers on experimental systems, whereas direct translational findings from humans remain limited. Future study must clarify tissue specificity, activation timing, relationships with physiological inflammatory processes including ovulation, and the potential for pathway manipulation to improve clinically important reproductive outcomes. Nevertheless, current studies support the view that cGAS-STING is not an ancillary route in reproductive disease, but rather a pivotal molecular interface that translates intracellular damage into infertility-related dysfunction.

## 8. Limitations and Future Directions

The existing data offers a consistent mechanistic framework; nonetheless, many limitations must be noted. Data are mostly derived from experimental models, including in vitro systems and animal research, where cellular activity is examined under controlled settings that may not completely replicate the intricacies of human reproductive physiology. Variations in the initiation and maintenance of the cGAS-STING signalling pathway may be influenced by species differences in ovarian dynamics, endocrine regulation, and immunological response. Reproductive physiology varies significantly between mice and humans, including ovarian cyclicity, follicular recruitment patterns, granulosa cell metabolic behaviour, and inflammatory signalling characteristics. Moreover, the molecular heterogeneity and endocrine responsiveness that are observed in human follicles, particularly during assisted reproduction and senescence, may not be completely replicated by granulosa cells derived from experimental animal models. Therefore, direct extrapolation of mechanistic findings from animal models to human reproductive ageing and IVF outcomes should be interpreted cautiously until larger translational and clinical studies become available.

A significant constraint is the diversity of reproductive diseases. Polycystic ovarian syndrome, endometriosis, and implantation failure are disorders characterised by diverse phenotypes, influenced by genetic predisposition, metabolic state, and environmental factors. The activation of cGAS-STING signalling is unlikely to be consistent across all patients. Targeted treatments will be contemplated only after the identification of certain subgroups that significantly influence this route. Neglecting to execute this classification jeopardises the nuanced understanding of the intricate pathophysiology.

The temporal dimensions of route activation remain poorly understood. Transient innate immune signalling may be physiologically significant in processes such as ovulation or tissue remodelling, but sustained activation contributes to disease. Distinguishing these phases in vivo is challenging and requires longitudinal examination rather than a single time-point evaluation. Comprehending the timing and length of cGAS-STING signalling in reproductive organs will be essential for its therapeutic significance.

Another domain requiring more investigation is the interplay with other signalling networks. Crosstalk exists across pathways related to oxidative stress, mitochondrial dynamics, autophagy, and hormonal signalling. But the order of these systems remains ambiguous. The dual function of cGAS-STING activation as both a main initiator and an enhancer of pre-existing dysfunction, depending upon environment. Therapeutic procedures will be refined and unexpected consequences mitigated by elucidating these linkages.

Future research should focus on a more systematic integration of molecular, metabolic, and clinical data. Integrating analyses of follicular fluid, granulosa cell profile, and reproductive outcomes may provide significant insights into how intracellular signalling correlates with clinical heterogeneity. The advancement of non-invasive biomarkers for pathway activation would enhance patient classification and monitoring. Future translational validation efforts must integrate prospective investigations that combine follicular fluid inflammatory profiling, granulosa cell transcriptome analysis, extracellular vesicle characterisation, and mitochondrial stress biomarkers with IVF laboratory and clinical results. Correlations between cGAS-STING-related molecular markers and fertilisation rates, embryo quality, implantation, and pregnancy outcomes may elucidate the clinical importance of pathway activation in human reproduction.

Moreover, validation across various reproductive illnesses and age demographics is necessary to ascertain if cGAS-STING activation constitutes a generic stress response or a disease-specific molecular profile. The standardisation of sampling procedures, scheduling of specimen collection, and molecular tests will be essential prior to any prospective clinical application or therapeutic targeting.

## 9. Conclusions

cGAS-STING signalling is emerging as a critical molecular interface between intracellular stress and reproductive dysfunction. Evidence from studies of ovarian, endometrial and systemic settings has supported a model in which mitochondrial instability, dysfunctional DNA management and chronic activation of innate immune pathways converge to influence cellular behaviour. This pathway, rather than an isolated inflammatory mechanism, ties together metabolic status, organelle integrity and genomic stability into a single response that has a direct impact on oocyte competence, follicular development and endometrial receptivity.

A consistent pattern is observed across different reproductive conditions. Similar downstream consequences of chronic signalling activation and loss of cellular coordination are triggered by different upstream signals, including metabolic imbalance, oxidative stress, environmental exposure and ageing-related changes. This convergence provides a mechanistic explanation for the overlap observed between disorders such as polycystic ovary syndrome, endometriosis, and implantation failure, and emphasises the importance of intracellular organization in maintaining reproductive potential.

The recognition of this pathway as a common molecular denominator offers new perspectives for both research and clinical practice. Approaches aimed at restoring mitochondrial function, improving cellular quality control, and modulating stress signalling may complement existing reproductive strategies. Further work is needed to define the clinical relevance of cGAS-STING activation in human fertility and to determine whether targeted interventions can translate into improved outcomes.

In general, the perspective of infertility as a problem of intracellular stress and DNA sensing moves the focus from individual clinical symptoms to a more holistic biological context. This view provides a starting point for future studies and promotes the evolution of more personalised and mechanism-focused strategies in reproductive medicine.

## Figures and Tables

**Table 1:** Core Molecular Components and Functional Dynamics of the cGAS-STING Signaling Pathway.

Component	Molecular Role	Upstream Trigger	Downstream Effect	Relevance to Reproduction
cGAS	Cytosolic DNA sensor	mtDNA leakage, ectopic nuclear DNA	cGAMP synthesis	Granulosa cell inflammatory activation and intracellular DNA sensing
STING	Intracellular signaling adaptor	cGAMP binding	TBK1, IRF3, and NF-κB activation	Increased inflammatory cytokine signaling and impaired steroidogenic activity, including reduced CYP19A1 expression
TBK1	Serine/threonine kinase	STING activation	IRF3 phosphorylation and interferon signaling	Promotion of inflammatory responses and stress-related transcriptional programs in follicular cells
NF-κB	Transcription factor	STING/TBK1 signaling	IL-6, TNF-α, and pro-inflammatory cytokine transcription	Follicular inflammatory signaling and granulosa cell stress responses
IRF3	Transcription factor	TBK1-mediated phosphorylation	Type I interferon gene activation	Innate immune activation within the ovarian and follicular microenvironment

Key molecular components of the cGAS-STING signalling pathway, their upstream activators, intracellular functions and downstream transcriptional effects. The interplay between DNA sensing and inflammatory signalling pathways in ovarian physiology is of particular interest. Accumulation of cytosolic DNA results in cytokine production, interferon signalling, and changes in granulosa cell function through coordination of cGAS, STING, and downstream mediators such as TBK1, IRF3, and NF-κB.

**Table 2:** Molecular Determinants of Oocyte Competence and IVF Outcomes Linked to cGAS-STING Activation.

Biological Alteration	Molecular Mechanism	Effect on Oocyte	IVF Outcome
Mitochondrial dysfunction	ROS ↑, mtDNA leakage	Spindle defects	Aneuploidy ↑
cGAS-STING activation	NF-κB, IFN signaling	GC inflammation	Poor oocyte quality
SASP phenotype	Chronic cytokine release	Altered microenvironment	Reduced fertilization
EV signaling changes	miRNA dysregulation	Gene expression shifts	Variable embryo quality
Follicular fluid imbalance	Cytokines/metabolites	Metabolic instability	Lower blastocyst rates
Endometrial activation	STING-mediated inflammation	Impaired receptivity	Implantation failure

This table demonstrates the relationship between molecular changes in the oocyte and their impact on oocyte quality and assisted reproductive outcome. Dysregulations in mitochondrial function, inflammatory signalling, extracellular vesicle communication and follicular fluid composition converge to influence oocyte maturation, fertilisation potential and embryo development. We define the cGAS-STING pathway as a pivotal mediator of cellular stress and clinically relevant IVF endpoints.

**Table 3:** Activation of cGAS-STING Signaling Across Reproductive Disorders: Molecular Mechanisms and Clinical Implications.

Condition	Model/Study Type	Mechanism	Key Finding	Clinical Implication
PCOS	Mouse (Zhao et al.), [100]	mtDNA leakage → cGAS-STING	GC apoptosis, inflammation	Reduced oocyte quality
Endometriosis	Human + in vitro (Zhu et al.), [26]	STING → autophagy	Increased invasion	Disease persistence
Adenomyosis	Human (Lin et al.), [15]	cGAS-STING ↑ cytokines	Dysmenorrhea correlation	Chronic inflammation
IUA	Human + mouse (Wu et al.), [88]	mtDNA → EMT	Fibrosis	Implantation failure
Endometritis	Human (Li et al.), [119]	LPS → mtDNA → STING	Cytokine increase	Reduced receptivity
Ovarian ageing	Mouse (Pan et al.), [91]	mtDNA → SASP	GC senescence	Decline in fertility

Summary of experimental and clinical evidence for the role of cGAS-STING signalling in major reproductive disorders. Each of them is defined by its molecular trigger, major mechanistic pathway and functional consequences. Mitochondrial dysfunction and cytosolic DNA accumulation are common upstream events in a variety of pathologies that lead to chronic inflammatory signalling, tissue remodelling, and altered reproductive function.

## Data Availability

No new data were created or analyzed in this study. Data sharing is not applicable to this article.
